# Supplementary material for: Developing the Pneumonia-Optimized Ratio for Community-acquired pneumonia: An easy, inexpensive and accurate prognostic biomarker
Source: PLoS One. 2021 Mar 23;16(3):e0248897. doi: 10.1371/journal.pone.0248897 (PMC7987181; doi:10.1371/journal.pone.0248897)

S1 Fig. Prognostic properties of CURB-65 in our sample. CURB-65 is an acronym for Confusion, Urea, Respiratory Rate, Blood pressure and 65 years-old.


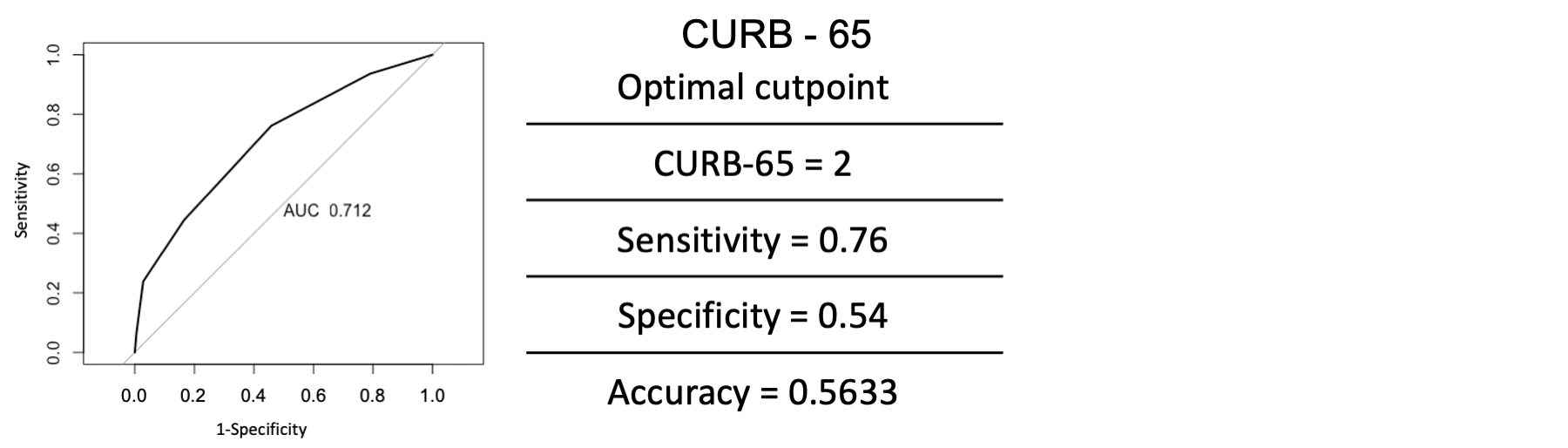

Supplement: S1 Fig — CURB-65 is an acronym for confusion, urea, respiratory rate, blood pressure and 65 years-old. (DOCX) [file pone.0248897.s002.docx]
